# Microdissection of Shoot Meristem Functional Domains

**DOI:** 10.1371/journal.pgen.1000476

**Published:** 2009-05-08

**Authors:** Lionel Brooks, Josh Strable, Xiaolan Zhang, Kazuhiro Ohtsu, Ruilian Zhou, Ananda Sarkar, Sarah Hargreaves, Robert J. Elshire, Douglas Eudy, Teresa Pawlowska, Doreen Ware, Diane Janick-Buckner, Brent Buckner, Marja C. P. Timmermans, Patrick S. Schnable, Dan Nettleton, Michael J. Scanlon

**Affiliations:** 1 Department of Plant Biology, Cornell University, Ithaca, New York, United States of America; 2 Plant Biology Department, University of Georgia, Athens, Georgia, United States of America; 3 Center for Plant Genomics, Iowa State University, Ames, Iowa, United States of America; 4 Cold Spring Harbor Laboratory, Cold Spring Harbor, New York, United States of America; 5 Division of Science, Truman State University, Kirksville, Missouri, United States of America; 6 Department of Plant Pathology, Ithaca, New York, United States of America; 7 Agriculture Research Service Department, United States Department of Agriculture, Washington, D.C., United States of America; 8 Department of Statistics, Iowa State University, Ames, Iowa, United States of America; The University of North Carolina at Chapel Hill, United States of America

## Abstract

The shoot apical meristem (SAM) maintains a pool of indeterminate cells within the SAM proper, while lateral organs are initiated from the SAM periphery. Laser microdissection–microarray technology was used to compare transcriptional profiles within these SAM domains to identify novel maize genes that function during leaf development. Nine hundred and sixty-two differentially expressed maize genes were detected; control genes known to be upregulated in the initiating leaf (P0/P1) or in the SAM proper verified the precision of the microdissections. Genes involved in cell division/growth, cell wall biosynthesis, chromatin remodeling, RNA binding, and translation are especially upregulated in initiating leaves, whereas genes functioning during protein fate and DNA repair are more abundant in the SAM proper. In situ hybridization analyses confirmed the expression patterns of six previously uncharacterized maize genes upregulated in the P0/P1. P0/P1-upregulated genes that were also shown to be downregulated in leaf-arrested shoots treated with an auxin transport inhibitor are especially implicated to function during early events in maize leaf initiation. Reverse genetic analyses of *asceapen1* (*asc1*), a maize *D4-cyclin* gene upregulated in the P0/P1, revealed novel leaf phenotypes, less genetic redundancy, and expanded D4-CYCLIN function during maize shoot development as compared to *Arabidopsis*. These analyses generated a unique SAM domain-specific database that provides new insight into SAM function and a useful platform for reverse genetic analyses of shoot development in maize.

## Author Summary

All the organs of plant shoots are derived from the shoot apical meristem (SAM), a pool of plant stem cells that are both organogenic and self-sustaining. These dual SAM functions take place in distinct yet adjacent meristematic domains; leaves are derived from the peripheral zone (PZ) of the SAM whereas cells lost during organogenesis are replenished from the central zone (CZ). Deciphering the global patterns of differential gene expression within these discrete SAM functional domains is integral toward understanding the molecular-signaling networks that regulate plant development. We utilized laser-microdissection technology to isolate tissues from the SAM crown and center (SAM-proper) and from initiating leaves (P0/P1) at the SAM periphery for use in microarray comparisons of gene expression within these SAM functional domains. Nine hundred and sixty-two maize genes were differentially expressed, confirming that the distinct functions of these meristematic domains involve widespread differences in gene expression. Genes involved in cell division, cell wall biosynthesis, chromatin structure, and RNA binding are especially upregulated in initiating leaves, whereas genes regulating protein stability and DNA repair are upregulated in the SAM proper. Mutations in a *D-cyclin* gene that was upregulated in the P0/P1 render narrow-leafed mutant plants with defective stomatal patterning, providing functional genetic data for a previously uncharacterized maize gene.

## Introduction

**Figure 1:**
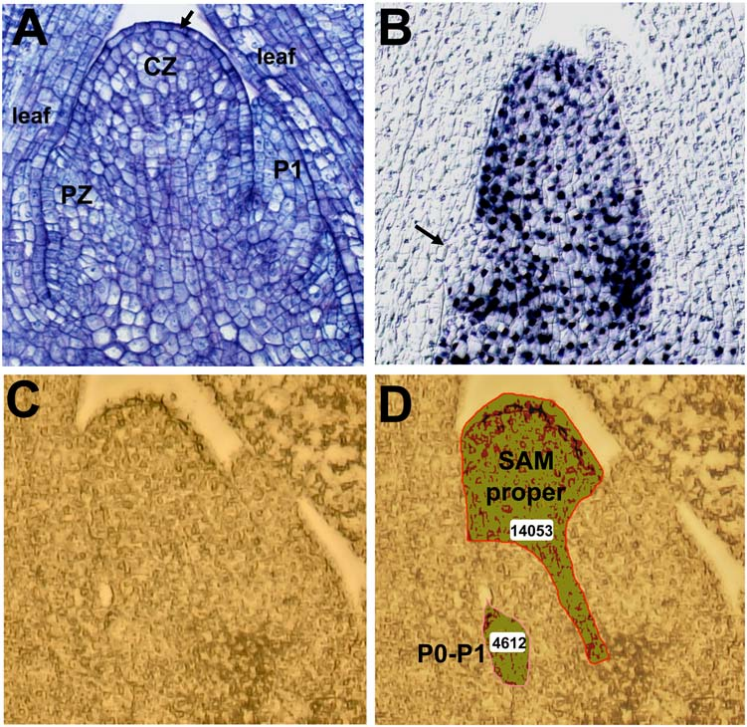
Domains in the maize SAM. (A) Toluidine blue O stained image of the maize seedling shoot apex showing the SAM surrounded by older leaf leaf primordia (leaf) and a newly-initiated leaf (P1). SAM histological zonation (CZ and PZ) and layering (L1 marked with arrow) are noted. (B) KNOX immunolocalization of the maize shoot apex. KNOX proteins (blue) accumulate in nuclei of the SAM and stem, but are excluded from the initiating P0/P1 leaf primordium (arrow) and older leaf primordia. (C–D) Micrograph of the maize shoot apex (C), and the SAM-proper and P0/P1 domains (outlined in D) captured by laser microdissection. Relative tissue areas comprising each domain are indicated in the white rectangles.

The maize shoot apical meristem (SAM) is a complex signaling network of distinct structural and functional domains that performs two essential developmental functions during plant shoot development: (1) self-maintenance and (2) organogenesis. Responsible for the development of all above ground organs in the plant, the SAM must maintain a precise equilibrium during which cells lost to newly-initiated leaves are replenished to maintain the SAM proper. Comprised of two tissue layers, the single-celled tunica (L1) and a multilayered corpus (L2), the maize SAM displays histological zonation that is correlated with its functions (Figure 1A). Determinate lateral organs arise from the peripheral zone (PZ) whereas the central zone (CZ) is comprised of more slowly dividing meristem initial cells that replenish the SAM. Although Caspar Wolff first recognized the SAM as the organogenic center of the plant shoot almost 250 years ago [1], detailed mechanisms of SAM function remain a fundamental question in plant biology.

Molecular genetic analyses have identified a growing number of genes contributing to the complexity of SAM function in maize. The homeobox gene *knotted1* (*kn1*) is required for meristem indeterminacy; null kn1 mutants fail to maintain the SAM [2],[3]. Down-regulation of KN1 accumulation in the PZ precedes lateral organ initiation, and is correlated with auxin transport and expression of the *knotted1-homeobox* (KNOX) regulator *rough sheath2* (*rs2*) in the PZ [4]–[7]. SAM size is also controlled by the cytokinin-inducible RESPONSE REGULATOR ABPHYL1, in which mutations increase SAM size and lead to disrupted phyllotaxy in the maize shoot [8],[9]. Maize leaves are formed via recruitment of ∼200 leaf founder cells from the PZ of the SAM [10], followed by differentiation along three developmental axes (proximo-distal/medio-lateral/adaxial-abaxial). Genetic analyses have identified several maize genes involved in these SAM functions, including those required for leaf initiation and phyllotaxy (*terminal ear1* and *aberrant phyllotaxy1*), proximodistal patterning (*rs2* and *semaphore1*), mediolateral development (*narrow sheath1&2*, *ragged seedling2*, *wavy auricle in blade1*), and adaxial-abaxial patterning (*rolled1*, *miR166*, *leafbladeless1*, *milkweed pod1*) [11], [8], [12]–[19]. Elucidation of the regulatory networks that coordinate these intersecting developmental functions will be bolstered by the use of genomic approaches to generate testable models for the SAM interactome, followed by comprehensive genetic and biochemical analyses to test and extend these hypotheses.

The complementary expression domains of the molecular markers *rs2* and *kn1* clearly illustrate that indeterminate cells of the SAM proper are immediately juxtaposed to leaf founder cells within the maize shoot apex (Figure 1B). The close proximity of these distinct functional domains presents technical barriers to comparative analyses of these discrete SAM functions. However recent technical advances have enabled a genomics approach toward the molecular dissection of SAM function. The relatively large size of the maize SAM, 50–250 founder-cells are recruited into the incipient leaf versus 25–30 in *Arabidopsis* [10],[20], renders the maize plant especially tractable to laser-microdissection technologies. Laser-microdissection permits the precise isolation of specific tissues, organs, or cells from fixed and sectioned plant tissues adhered to microscope slides [21]. Nanogram quantities of RNA extracted from less than 1 mm^2^ of microdissected tissue (comprising five to ten whole SAMs) can be linearly amplified using T7 RNA polymerase to generate microgram quantities of RNA sufficient for transcriptional profiling using microarray technology [22]–[26]. Owing to its unique ability to sample discrete microdomains in plant tissues, laser-microdissection eliminates the transcriptional noise contributed by adjacent or contaminating unrelated tissues and thereby enables transcriptional profiling that is focused on the cells and tissues of interest.

Laser microdissection-microarray technology was utilized in comparative transcriptional analyses of functional domains in the maize SAM. Gene expression within SAM microdomains encompassing the initiating maize leaf (P0/P1) and the stem cells of the SAM-proper was analyzed; 962 maize genes were differentially expressed in this comparison. Control genes of known expression domain confirmed the accuracy of the laser microdissections and validate the dataset. Genes predicted to function during cell division/growth, chromatin remodeling, RNA-binding, cell wall biosynthesis and translation are especially upregulated in initiating leaves, whereas genes involved in protein fate and DNA repair are prevalently expressed within the SAM-proper. In situ hybridization analyses, and qRT-PCR analyses of apices that are arrested in leaf initiation identified twelve maize genes predicted to function during leaf initiation. Reverse genetic analyses of the maize D-cyclin gene *asceapen1* (*asc1*) confirmed its predicted function during maize leaf and shoot development; novel mutant phenotypes revealed differing levels of genetic redundancy and divergent patterns of subfunctionalization among *cyclin* paralogues in maize and *Arabidopsis*. Our data provide a unique database that provides insight into SAM function and a useful platform for reverse genetic and biochemical analyses of maize shoot development.

## Results

### Laser Microdissection–Microarray Analyses of SAM Domains

Maize seedlings were grown under controlled conditions and processed for laser microdissection of SAM domains (see Materials and Methods). Although KNOX immunohistolocalization analyses clearly delineate the leaf/non-leaf boundary in the maize shoot apex (Figure 1B), these treatments require crosslinking fixatives that preclude the extraction of RNA from microdissected tissues. Therefore, in lieu of molecular makers, two distinct SAM domains were captured using morphological/anatomical cues (Figure 1 C–D). The “SAM-proper” comprised the apical crown and central stem of the shoot apex, and is estimated to include the CZ. Tissue extracted from the “P0/P1” domain included the PZ and the newest-initiated lateral organ that formed a protruding buttress on the SAM flank. Care was taken to avoid the SAM peripheral zone during captures of the SAM-proper domain; likewise the P0/P1 samples were harvested from a depth of no more than three cell-layers in order to avoid tissues that typically accumulate KNOX proteins (Figure 1B), markers of meristematic identity [2]. Tissues derived from ten total SAMs were pooled into domain-specific samples comprising a single biological replicate.

Following RNA extraction and amplification (see Materials and Methods), six such biological replicates were utilized in microarray hybridizations to 29,600 total elements (including approximately 23,000 unique maize genes) contained on the customized maize cDNA microarrays SAM1.1 and SAM3.0 [described in 25],[26]. Replete with genes identified from meristematic tissues, SAM 1.1 contains over 7,500 cDNAs derived from maize inflorescences and SAM 3.0 contains over 10,500 cDNAs derived from vegetative apices (i.e. SAM plus four leaf primordia). For each array platform, three of the six cDNA pairs were labeled with Cy3 from the SAM-proper and Cy5 for the P0/P1. Dye assignments were reversed for the other three replications. Normalized Cy5 and Cy3 signals were used to test for evidence of differential expression among the SAM domains using a linear model analysis for each gene (see Materials and Methods).

**Figure 2:**
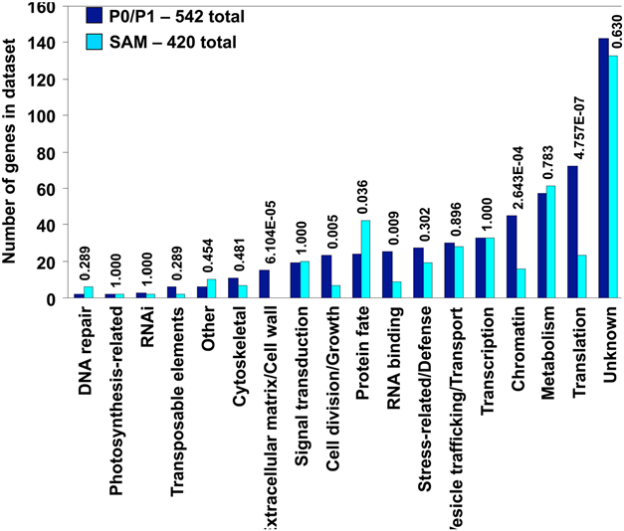
Differential gene expression in SAM domains. Laser microdissection microarray analyses identified 962 maize genes, separated into 18 different functional categories, as differentially expressed in the SAM-proper and P0/P1 domains. *P*-values of significance for SAM domain-specific differential expression of gene categories are indicated.

A total of 1,312 array elements were differentially expressed in these SAM domains utilizing cut-off parameters of *P*-value<2.93 E-4 and fold change>2.0. Alignment of these 1,312 cDNA sequences to predicted genes within the sequenced maize genome (see Materials and Methods) identified 962 maize gene contigs (MGC) that were differentially expressed in the SAM-proper and P0/P1 leaf primordia (Table S1). These included 542 genes upregulated in the P0/P1 leaf and 420 genes upregulated in the SAM-proper (Figure 2). Notably, 48 (i.e. 3.6%) of the 1,312 differentially expressed array elements did not align to any sequenced MGC; the EST accession numbers of these unaligned genes are thus listed among the 962 genes contained in Table S1. None of these unaligned EST sequences are predicted to comprise repetitive retrotransposons, but presumably correspond to a portion of the maize gene space that is as yet unrepresented in the sequenced portion of maize genomic DNA. Approximately 31.8% of the cDNA array dataset aligned with equal affinity to multiple MGCs, and are likely to comprise gene family members for which the available cDNA sequence does not distinguish between close paralogs (Table S1).

The estimated false discovery rates were 1.1% for SAM1.1 and 0.5% for SAM3.0. Bioinformatic predictions of function were performed for all differentially expressed genes as described [27], and are presented at the *SAM-The Maize Shoot Apical Meristem Project* database created during this project (http://sam.truman.edu/geneva/geneva.cgi). A total of eighteen different Gene Ontology (GO) functional categories are identified as detailed below (Figure 2), including: DNA repair; photosynthesis related; RNAi; transposable element; other; cytoskeletal; extracellular matrix/cell wall; signal transduction; cell division/growth; protein fate; RNA binding; stress related/defense; vesicle trafficking/transport; transcription; chromatin; metabolism; translation; and unknown.

### Control Genes Exhibit SAM Domain-Specific Expression

Control genes whose expression in either the SAM-proper or the P0/P1 is described previously attested to the precision and accuracy of the SAM domain microdissections (Figure 1D; Table 1 and references therein). For example, the meristem maintenance gene *knotted1* ^1^ (*kn1*; [2], see Dataset S1 for a list of the MGC accession numbers corresponding to superscripted numerals in this text), the phyllotaxy regulator *terminal ear1* ^2^ (*te1*; [11]), the trans-acting siRNA (tasiRNA) biogenesis gene *leafbladeless1* ^3^ (*lbl1*; [28]) and the maize homolog of the sterol biosynthetic gene *fackel* ^4^ [29] were all identified in our microarrays as up-regulated in the SAM-proper, in agreement with published expression analyses. Likewise, the *knox*-regulatory gene *rough sheath2* ^5^ (*rs2*; [6],[7]), a maize homolog of *growth-regulating factor1* ^6^ (*grf1*; [30]), a maize *auxin response factor5/monoteros1* ^7^ (*arf5/monopteros*; [31]), and several members of the *yabby* gene family of transcription factors (*yab15* ^8^, *yab10* ^9^; *Zm-drooping leaf-like* ^10^; [16],[26]) were all up-regulated in the P0/P1 domain, as predicted from previous studies. Control genes also up-regulated in the initiating leaf included maize orthologues of the auxin transporters *pinformed1* ^11^ (*pin1*) and *auxin insensitive1* ^12^ (*aux1*), as well as the cell wall-loosening gene *beta expansin8* ^13^ (*expb8*), all of which are known to be expressed during leaf initiation in maize and/or *Arabidopsis* [32]–[35].

**Table 1:** Microarray expression data for selected SAM domain control genes.

Maize Contig	P-value	Fold change	Gene prediction	Function
AC191426.2-Contig19	0.000167	2.52; SAM	*knotted-1*	SAM maintenance
AC200561.4-Contig49	7.21e-07	5.67; SAM	*leaf bladeless*	adaxial patterning
AC214821.2-Contig11	3.99e-05	2.78; SAM	*terminal ear1*	phyllotaxy
AC183520.3-Contig25	8.06e-05	2.43: SAM	*Zm-fackel*	sterol biosynthesis
AC185600.3-Contig17	6.61e-06	8.22; P0/P1	*rough sheath2*	KNOX regulation
AC210607.2-Contig20	7.82e-06	3.62; P0/P1	*Zm-pin1*	auxin efflux
AC177947.2-Contig69	2.15e-07	6.57; P0/P1	*Zm-aux1*	auxin influx
AC190645.3-Contig32;	1.17e-08	20.37; P0/P1	*Zm-growth-regulating factor1*	cell expansion
AC204518.4-Contig32	1.98e-06	5.01; P0/P1	*Zm-monopteros1*	transcription
AC202451.2-Contig53	6.07e-05	3.73; P0/P1	*Zm-yabby10*	transcription
AC194098.3-Contig35	5.74e-06	16.09; P0/P1	*Zm-drooping leaf*	transcription
AC195538.2-Contig93	1.38e-09	11.97; P0/P1	*Zm-yabby15*	transcription
AC204864.2-Contig55	5.62e-05	2.73; P0/P1	*Zm-beta expansin8*	cell-wall loosening

### Differential Gene Expression in SAM Microdomains

Microarray analyses of the SAM-proper and P0/P1 apical domains reveal discrete GO functional categories of preferentially expressed genes (Figure 2; Table S1). For example, significantly more (*P*<0.036) genes involved in protein fate/ubiquination were found to be upregulated in the SAM-proper (42) as compared to the P0/P1 (24), including multiple paralogues encoding a predicted E2 UBIQUITIN CONJUGATING ENZYME-LIKE^14^. Also, three-fold more DNA-repair genes were upregulated in the SAM-proper than in the P0/P1, including maize orthologues of *rad23* ^15^, *radA* ^16^, *mus1* ^17^ and the *SNF2 domain/helicase protein* ^18^.

Genes comprising five predicted functional categories were significantly upregulated in the P0/P1, including those functioning in the extracellular matrix/cell wall (*P*<6.10E-05), cell division/growth (*P*<0.005), RNA binding (*P*<0.009), chromatin (*P*<2.64E-04), and translation (*P*<4.76E-07). Fifteen genes involved in cell wall biology were upregulated in the P0/P1, whereas none were upregulated in the SAM-proper. These included genes encoding cell wall GLYCOPROTEINs^19^ and GLYCOSYLASES^20^, an ALPHA-EXPANSIN^21^, a BETA-EXPANSIN^13^, and a CELL WALL-ANCHORED PROTEIN^22^.

Differentially expressed genes encoding proteins involved in cell division and growth in the P0/P1 outnumbered those identified in the SAM-proper twenty-three to seven (Figure 2). Reflecting the increased mitotic activity found in the peripheral zone and initiating leaf as compared to the SAM central zone, these included genes encoding various CYCLINs^23–27^ and a putative maize homolog of mammalian *growth regulating factor1* ^6^. Also identified are at least four maize paralogs of the TRANSLATIONALLY-CONTROLLED TUMOR PROTEIN (TCTP^28–31^), guanine exchange factors that control organ size in *Drosophila* and mammals by regulating a specific dRheb-GTPase within the target of rapamycin (TOR) signaling pathway [36]. Recent analyses of a *TCTP* gene in *Arabidopsis* revealed increased expression in rapidly growing tissues; reverse genetic mutant plants exhibited a range of developmental defects including reduced cell size and leaf expansion, and decreased sensitivity to auxin [37].

**Figure 3:**
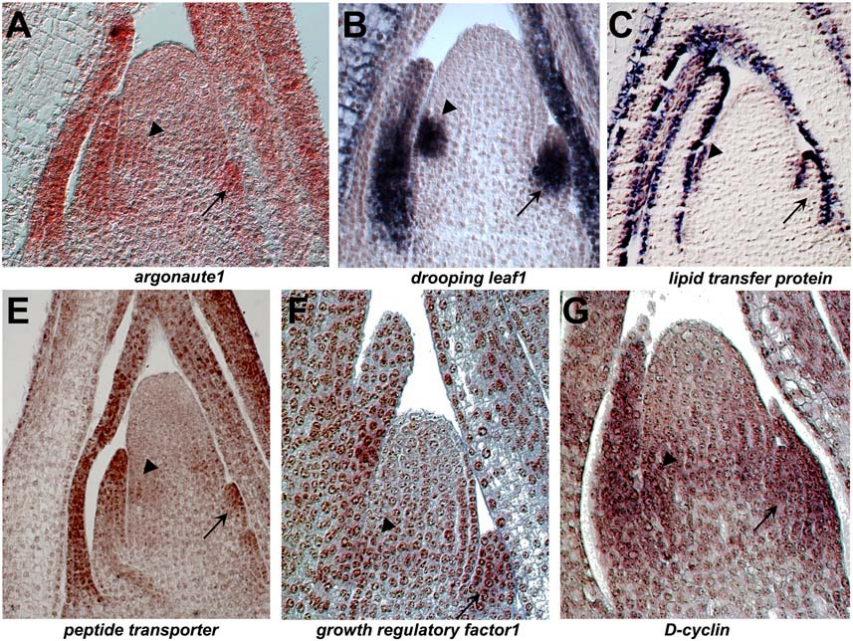
In situ hybridization analyses of genes upregulated in the P0/P1. Transcripts for six genes identified in SAM microarray analyses as differentially expressed in the P0/P1 SAM all accumulate in young leaf primordia (arrows) and in the lower SAM periphery and P0 (arrowhead).

Three distinct *argonaute1*-like maize paralogues^32–34^ were identified in our microarray data, all of which were upregulated in the P0/P1 (Table S1). *Arabidopsis* contains two close paralogues, *argonaute1* (*ago1*) and *pinhead1/zwille1* (*pnh1/zll1*), which encode components of the multi-subunit RNA-induced silencing complex (RISC; [38]). In keeping with their partially overlapping roles in the miRNA-regulated control of leaf polarity and of SAM maintenance, *ago1* is evenly expressed throughout the *Arabidopsis* SAM and young leaf primordia [39]–[42], whereas *pnh1/zll1* transcripts accumulate preferentially in leaf primordia and in the vasculature [43]–[45]. Owing to the nearly identical amino acid sequences of AGO1 and PNH1, it is not possible to predict which of maize *ago*-like genes are *ago1* orthologues and which are *pnh1/zll1* orthologues. However, in situ hybridization analysis of a maize *ago1*-like gene that was upregulated more than seven-fold in the P0/P1 confirmed our microarray data, and revealed a *pnh1/zll*-like expression pattern (Figure 3A). Although transcripts are indeed detected in the SAM crown and center, more abundant transcript accumulation is observed in the leaf founder cells, the SAM periphery, and in young leaf primordia (Figure 3A).

In contrast to the miRNA regulatory *ago1* genes identified in the P0/P1, the tasi-RNA gene *lbl1* ^3^ [28] and the siRNA effector protein gene *argonaute4* ^35^ (*ago4*; [46],[47]) were upregulated in the SAM-proper. Moreover, significantly more RNA-binding genes were preferentially expressed in initiating maize leaves compared to the SAM-proper, including four genes predicted to encode RIBONUCLEOPROTEINs^36–39^, numerous GLYCINE-RICH RNA-BINDING PROTEIN^40–44^ paralogs, and a maize homolog of the *Arabidopsis* flowering-time regulator *flowering locus K* ^45^ gene (*flk*; [48]; Table S1).

A preponderance of gene elements predicted to function during chromatin structure and remodeling were upregulated in the P0/P1 versus the SAM-proper (45 versus 16, respectively; Figure 2). For example, a *cytosine-5-methyltransferases* ^46^ and three *methyl-CpG DNA binding domain* ^47–49^ genes are specifically upregulated in the P0/P1. Likewise, whereas *hdt3–like* ^50^ and *sir2-like* ^51^ *histone deacetylase* gene are upregulated in the leaf initials, a *hdt2-like histone deacetylase* ^52^ is highly expressed in the SAM-proper and three *swib-domain* ^56–58^ gene paralogs are detected only in the SAM-proper.

Although the number of putative transcription factors (TFs) preferentially expressed in either the SAM-proper or the P0/P1 is exactly equal at thirty-three each (Figure 2), each SAM domain exhibited upregulation of various distinct TF genes not identified in the other. For example, the founding member of the *knotted-like homeobox* (*knox*) gene family *kn1* ^1^ is differentially expressed in the SAM-proper, while the related *knox* gene *gnarley1* ^59^ (*gn1*) is upregulated in the P0/P1. Although a previous report detected *gn1* expression in the shoot apex [49], the SAM domain specificity of *gn1* expression was not described previously. Likewise, a maize homologue of the *Arabidopsis leunig co-repressor* ^60^ [50] gene is identified in the SAM-proper, as were three paralogs encoding *B3-domain* ^61–63^ TFs. Developmental regulators of embryo and meristem development, many B3-domain TFs are shown to function via interaction with auxin or ABA signaling pathways [51],[52, and references therein]. In contrast, *rs2^5^*, *auxin response factor2* ^64^ (*arf2*), and multiple members of the *yabby* ^8–10^ gene family were identified in the P0/P1. RS2 represses *knox* gene expression in developing leaves [6],[7], whereas *arf2* accumulates in *Arabidopsis* lateral organs [53] and maize *yabby* genes are transcribed in the P0 and leaf primordia (Figure 3B; [16],[17],[26].

Lastly, the largest single gene category identified in our microarray analyses comprised genes of unknown predicted function, which contained 142 genes upregulated in the SAM-proper and 133 genes in the P0/P1 (Figure 2).

### Expression of SAM Domain-Specific Paralogs Reveals Gene Subfunctionalization

Distinct gene paralogs of the histone-methylating SET DOMAIN-encoding gene^53–55^ family are upregulated in the immediately adjacent apical domains that comprise the P0/P1 and SAM-proper. In addition, paralogs of six other maize gene families including *auxin response factor1* ^65, 66^ (*arf1*), *histone3* ^67, 68^, *histone4* ^69, 70^, *ubiquitin-conjugating enzyme E2* ^71, 72^, *ADP-ribosylation factor/Secretion-associated and Ras-related* ^73, 74^ protein (*ARF/SAR*), and the ubiquitin-ligase subunit gene *S-phase kinase-associated protein1* ^75, 76^ (*skp1*) exhibit preferential expression within the SAM-proper and the P0/P1, and thus provide intriguing evidence for subfunctionalization of these gene families within discrete functional zones of the maize SAM.

### Verification of Preferential Gene Expression via In Situ Hybridization

Focusing on previously uncharacterized maize genes implicated during leaf development, six genes upregulated in the P0/P1were subjected to in situ hybridization analyses in order to verify the domain-specific transcript accumulation predicted from our microarray data and identify novel patterns of gene expression. In addition to the maize *ago1*-like gene described in Figure 3A, five genes whose functions are yet to be demonstrated in maize were analyzed. These included a putative *oligopeptide transporter* ^77^, a *yabby* gene *drooping leaf1* ^10^, a predicted *growth-regulating factor* ^6^, a *lipid-transfer protein* ^78^, and a *D4-class cyclin* ^25^. In all cases the pattern of transcript accumulation observed in the in situ hybridizations correlated with the microarray data. Stronger signals were observed in the SAM periphery, P0, and small leaf primordia as compared to the SAM crown and center (Figure 3), which verified the P0/P1 upregulated expression observed in our microarray analyses.

### Cross-Reference Analyses of Leaf-Arrested Shoots: Identification of Genes Functioning during Early Leaf Initiation

**Figure 4:**
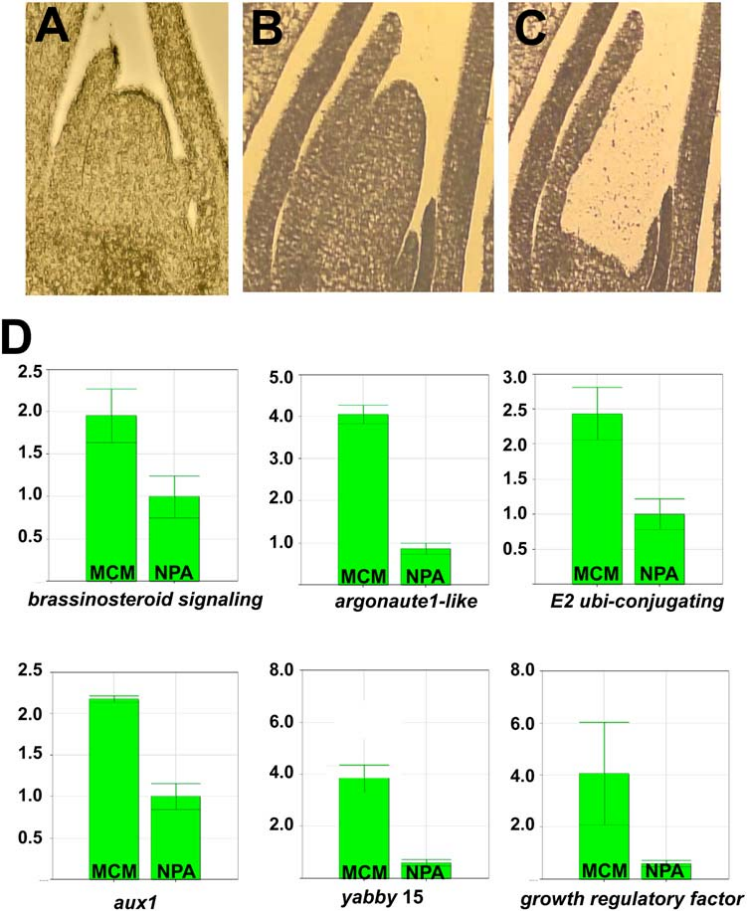
Gene expression in leaf-arrested SAMs. Maize shoot apices cultured in the absence (A) or in the presence of the auxin transport inhibitor NPA (B) were laser microdissected (C) and used in qRT-PCR analyses of candidate genes to identify six genes (D) downregulated in NPA-treated, leaf-arrested SAMs. Note the elongated, pin-shaped morphology of the NPA-treated SAM (B) that fails to initiate new leaf primordia.

Auxin transport is the earliest-demonstrated prerequisite to KNOX downregulation and leaf initiation from the SAM flank; disruption of auxin transport by the chemical inhibitor N-1-naphthylphthalamic acid (NPA) leads to the arrest of lateral organogenesis in plant shoots [54],[4],[5]. Therefore, NPA-induced inhibition of leaf initiation provides a compelling experimental system with which to monitor SAM gene expression during very early events in leaf development. Toward this end, 14-day-after-germination seedling shoot apices were dissected to remove all organs except the SAM and the six youngest leaf primordia and placed in tissue culture with or without 30 mM NPA as described [4]. As shown in Figure 4A–B, NPA-cultured SAMs became greatly elongated but failed to initiate any new leaves, whereas equivalent sibling apices generated 6–7 new leaf primordia in NPA-free culture. After 14 days in culture, samples were processed for SAM laser-microdissection mediated qRT-PCR analyses as described [55]. Genes found to be upregulated during leaf initiation but down-regulated during NPA-induced arrest of organogenesis are especially implicated to function during early stages of maize leaf development.

Transcript accumulation analyses were performed for nine genes that were significantly upregulated in the P0/P1 in our microarray analyses (Table S1). As shown in Figure 4D, qRT-PCR analyses revealed that transcripts of six of these nine genes were also down-regulated in leaf-arrested apices, including a second maize *ago1-like* ^32^ gene, a putative *brassinosteroid response factor* ^79^ gene, an *E2 ubiquitin-conjugating*-like^71^ paralog, the *aux1* ^12^ auxin transporter gene, a *yabby 15* ^8^ gene, and the *growth-regulating factor1* ^6^. One gene (a putative *seven-in-absentia*-like *ubiquitin ligase* ^80^) was weakly down-regulated in NPA-treated apices, whereas two genes (a *tctp-like* ^31^ gene paralog and a maize *AMP-dependent synthetase* ^81^) were unchanged in NPA-treated versus untreated shoots. Thus, six genes identified as upregulated in the P0/P1 are downregulated in shoot apices that are arrested in leaf initiation. We speculate that the three genes whose expression levels were unchanged following NPA treatment may mark a domain within the PZ that functions upstream or independent of auxin transport during leaf initiation, since accumulation of some PZ markers has been shown to persist in *Arabidopsis* pin1 mutants and in tomato apices treated with NPA [54]. Alternatively, these NPA-unaffected genes may not be preferentially expressed during early leaf initiation.

### Reverse Genetic Analysis Uncovers a Novel Gene Function during Maize Development

**Figure 5:**
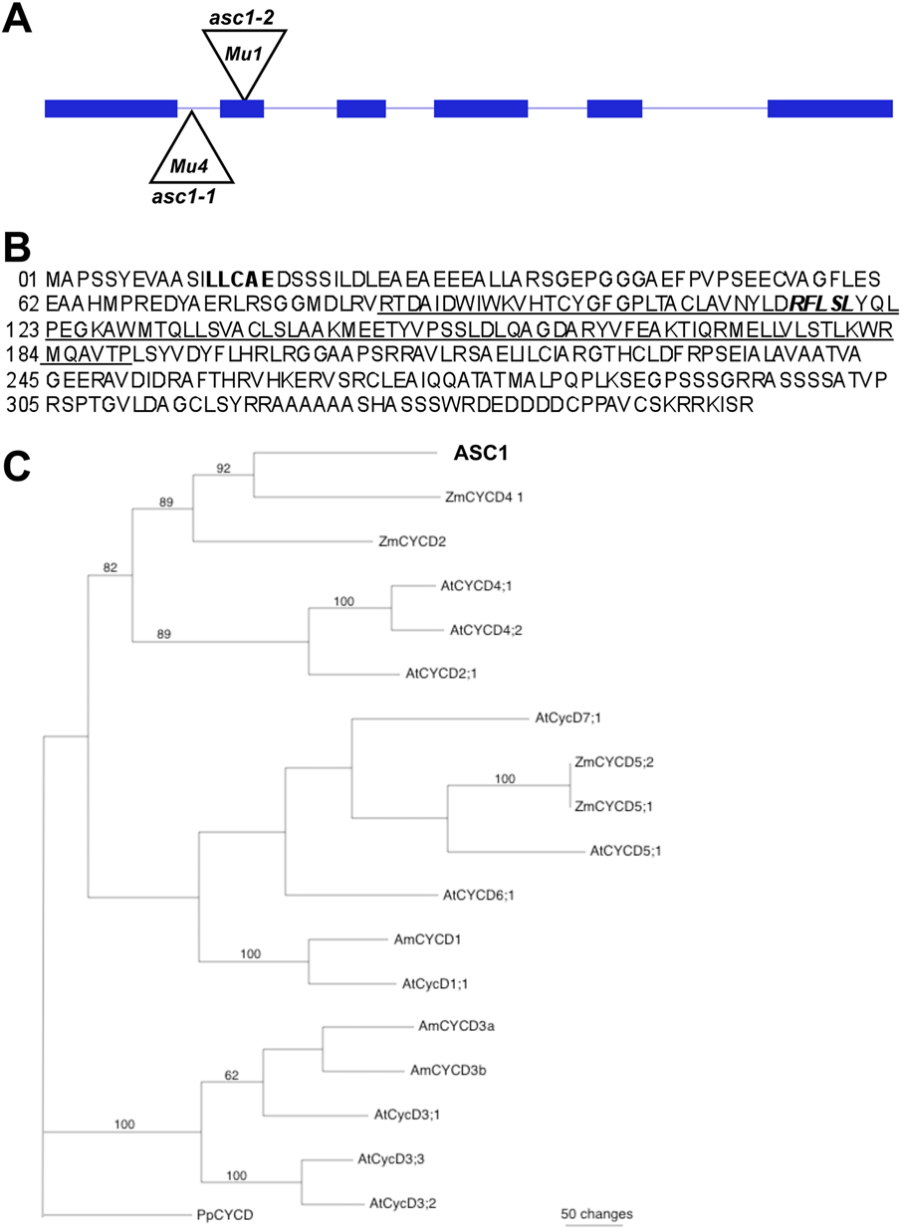
Gene structure and phylogenetic analysis of *asceapen1* (*asc1*). (A) The maize leaf *D4-cyclin* (*asc1*) gene is comprised of six exons (blue) and encodes a predicted ORF of 1068 base pairs. The locations of the insertion sites of *Mu* transposons (triangles) in asc1 mutant alleles are indicated. (B) Predicted 355 amino acid sequence of ASCEAPIN1. Sequences in bold denotes the conserved RETINOBLASTOMA-interacting domain. The underlined motif denotes the predicted CYCLIN BOX, and the sequences in bold italics correspond to the predicted CYCLIN recognition site. (C) Neighbor Joining phylogenetic analysis of selected D-CYCLIN proteins from maize, *Arabidopsis* and *Antirrhinum*, using a D-CYCLIN from the moss *Physcomitrella patens* as outgroup. Bootstrap values were obtained using 1000 replicates.

The differential gene expression data presented in this study identify genes implicated in SAM domain-specific functions during maize shoot development. Validation of these predicted functions, however, requires biochemical or genetic analyses. A reverse genetic strategy was implemented (see Materials and Methods) to investigate the function of a *D4-class cyclin* ^25^ gene that was identified as upregulated in the leaf primordia, which we have named *asceapen1* (*asc1*). In situ hybridization of seedling shoot apices verified the P0/P1 upregulated *asc1* expression observed in the microarray analyses (Figure 3E). The *asc1* gene contains six exons (Figure 5A) and is located at position 39,743–41,597 of contig 45 on maize chromosome 7. The 1068 bp open reading frame is predicted to encode a protein of 355 amino acids, which contains the canonical LxCxEx RETINOBLASTOMA-interaction domain that is characteristic of D-CYCLINS (Figure 5B;Wang et al., 2004). In addition, ASC1 contains the conserved amino cyclin box and a CYCLIN recognition motif. Limited expression profiles are described for four related maize *D-cyclin*s (including a *D2-cyclin*, a *D4-cyclin*, and two *D5-cyclins* [56], although expression within the vegetative SAM was not examined. No genetic analyses of D-CYCLIN function have been performed previously in maize.

D-CYCLINS perform an evolutionarily conserved growth-regulatory function to regulate progression through the G1 phase of the cell cycle [57]. Genetic analyses in *Arabidopsis* suggest that D-CYCLINS function as important regulators of asymmetric cell division, a process that is critical to developmental differentiation and has played a pivotal role in the evolution of multicellularity [reviewed in 58],[59]. *Arabidopsis* has 10 *CYCD* genes comprised of six subgroups (*CYCD1*, *CYCD2*, *CYCD4* (2 genes), *CYCD3* (3 genes), *CYCD5*, *CYCD6*, and *CYCD7* [60]. Overexpression analyses suggest that as a group, D-CYCLINS may regulate the developmental progression from cell proliferation to differentiation [61],[62],[63]. Genetic analyses reveal redundant functions for the three CYCD3 genes in *Arabidopsis* [64]. Single CYCD3 mutations yield non-mutant phenotypes; triple mutations condition small yet fertile plants with narrow leaves, a small SAM, and decreased cytokinin response. *CYCD4;1* is expressed in both shoot and root apices, although *CYCD4;2* is not detected in the SAM. Single mutations in *CYCD4;1* and *CYCD4;2* render no macrophenotype, although reduced numbers of anatomically normal stomata develop in mutant hypocotyls [65].

A phylogenetic analysis was performed on the maize ASC1 protein and thirteen additional plant D-CYCLINS for which transcriptional analyses and/or genetic analyses are documented [66],[56],[67], including four additional maize D-CYCLINS, ten *Arabidopsis* D-CYCLINs, and three D-CYCLINs from *Antirrhinum majus*. Utilizing the D1-CYCLIN from the moss *Physcomitrella patens* as an outgroup. ASC1 was placed on a well-supported clade together with the D4-CYCLINS and D2-CYCLINS from *Arabidopsis* (Figure 5C). All the other D-CYCLIN proteins were placed on separate clades; the D3-CYCLINS from *Arabidopsis* and *Antirrhinum* comprise a well-supported separate clade from ASC1. Reverse genetic analyses of *asc1* were instigated in order to investigate the function of this D4-CYCLIN in maize.

F2 seedlings were obtained from self-pollination of over 3,000 maize plants with *Mutator* (*Mu*) transposon activity, a maize transposon with an unusually high forward mutation rate [68]. A PCR-based reverse genetic strategy similar to previously published protocols ([69],[70]; see Materials and Methods) identified two independently-segregating *Mu*-insertion alleles of the *asc1* gene, designated *asc1-M1* and *asc1-M2* (Figure 5). The *asc1-M1* allele harbors a *Mu4* insertion in position 47 of the 129 bp intron 1, whereas the predicted null *asc1-M2* allele harbors a *Mu1* at position 31 of the 87 bp second exon (Figure 5A). RNA gel-blot hybridization analyses reveal that *asc1* transcript accumulation is greatly diminished in *asc1-M1* homozygotes and is virtually absent in *asc1-M2* homozygous plants, relative to non-mutant siblings (Figure S1).

**Figure 6:**
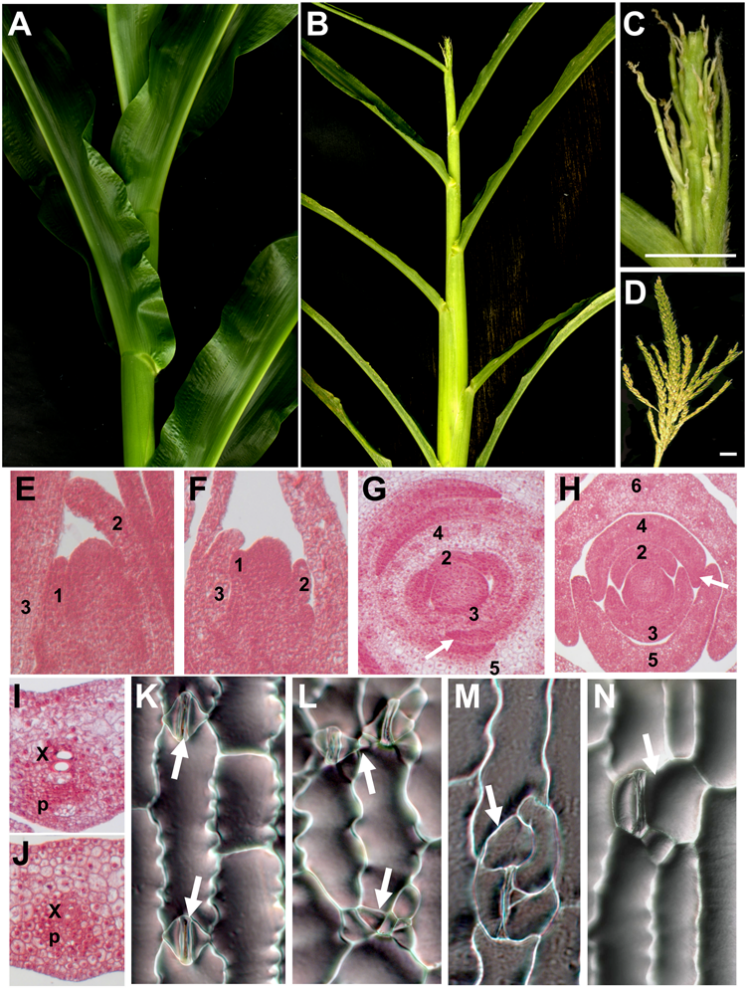
Developmental phenotypes of asc1 mutants. (A) Leaves of non-mutant maize leaves are much wider than the (B) narrow leaves formed on the short plants of asc1-2 mutants. The male inflorescence (tassel) of asc1-2 mutants form small, sterile branches devoid of florets (C), whereas non-mutant tassels are larger, and the numerous branches form multiple, pollen-bearing florets (D). Longitudinal sections of the shoot apex of (E) non-mutant and (F) asc1-2 mutant seedlings reveal a greatly reduced SAM size in the mutant apex. Transverse sections reveal the narrow leaf asc1 mutant phenotype. Note that the margins (white arrow) of the fourth numbered leaf completely overlap the shoot apex in non-mutant seedlings (G), but are severely truncated in the asc1-1 mutant (H). Development of leaf vascular bundles is also disrupted in asc1 mutant seedlings. The number and size of the xylem (x) and phloem (p) elements in the midvein of the sixth non-mutant leaf (I) are diminished in equivalent leaves of asc1-1 mutant seedlings (J). As compared to non-mutant siblings (K), major defects in stomatal (arrows) patterning and spacing are observed in the leaf epidermis of asc1-1 mutants (L), asc1-2 mutants (M), and in mutant progeny obtained from crosses between *asc1-M1* and *asc1-M2* heterozygotes, which fail to complement.

F2 progeny of plants heterozygous for *asc1-M1* or *asc1-M2* each segregate for short, infertile plants with very narrow leaves (Figure 6A–B). These mutant phenotypes co-segregate with homozygosity for *asc1* mutations, and interallelic crosses of plants heterozygous for *asc1-M1* and *asc1-M2* fail to complement (Figure 6N). No female inflorescences (ears) are observed in homozygous *asc1* mutant plants, and male inflorescences form only rudimentary tassels with sterile branches and no floral morphogenesis (Figure 6B–D). Histological examinations of asc1 mutant seedlings reveal extremely narrow leaves and small vascular bundles with reduced numbers of xylem and phloem vessels, as well as reduced SAM size (Figure 6E–J). Both mutant alleles conditioned equivalent phenotypes, although the range of phenotypes is more severe in plants homozygous for the exon-insertion allele *asc1-M2*.

Especially striking are the effects of *asc1* mutations on stomatal patterning and anatomy. Comprised of two subsidiary cells that surround and appress two smaller guard cells, interspaced stomatal complexes are formed via a series of ordered, asymmetric cell divisions in the leaf epidermis(Figure 6K; reviewed in [71]). Analyses of the asc1 mutant leaf epidermis reveal irregular stomatal patterning (Figure 6L–N). Two mutant stomatal complexes often form immediately adjacent to one another, a pattern not observed in non-mutant leaves. Other abnormalities include enlarged, distorted, and supernumery subsidiary cells and guard cells, which often develop immediately adjacent to completely normal stomatal complexes.

## Discussion

### A Unique Expression Database and a Platform for Future Analyses of SAM Function

Novel transcriptomic comparison of the functionally distinct microdomains within the maize SAM are presented, an analysis that was enabled by the relatively large size (∼120 µm) of the maize SAM as compared to *Arabidopsis*. The differential expression of 275 unknown genes within a particular SAM domain thereby provides a first suggestion of their potential function. Moreover, the documentation of seven instances wherein gene paralogues exhibited subfunctionalized preferential expression within distinct SAM microdomains provides insight into the evolution of specific gene families in maize. The entirety of this unique expression database is publicly available (http://sam.truman.edu/geneva/geneva.cgi) and represents a starting point for subsequent reverse genetic analyses of SAM function in maize. In addition, these genomic analyses are likely to uncover genes whose functions that are not amenable to traditional genetic analyses, owing to the embryo/seedling lethality that may result from mutations in genes required for early events in SAM ontogeny and/or leaf initiation.

### SAM Domains Exhibit Distinct Transcriptional Profiles That Correlate with Their Function

Genes whose SAM domain-specific expression are previously described in maize or *Arabidopsis* (Table 1) served as experimental controls for the analyses presented here, and attest to the power and precision of laser-microdissection for analysis of transcript accumulation within plant microdomains. Three distinct paralogs of *ago1* ^32–34^ are identified, each of which was upregulated in the P0/P1. *Arabidopsis* contains two *ago1*-like genes, one of which (*pnh1/zll1*) is preferentially expressed in leaf primordia whereas the other (*ago1*) is evenly expressed throughout the SAM and young leaves [39]–[45]. Although the extreme amino acid conservation observed in PNH1 and AGO1 precludes the identification of specific maize orthologues from homology alone, *in situ* hybridization analysis verified the leaf-preferential expression of one maize *ago* gene (Figure 3A) whereas a separate *ago1* paralog was downregulated in NPA-treated apices that are arrested in leaf initiation (Figure 4D). Our results are analogous to the reported leaf-upregulated expression of the rice *pinhead/zwille* orthologue *OsPNH1* [72]. We speculate that maize co-orthologs whose expression domains mirror that of the *Arabidopsis ago1* gene would not be identified in our microarray analyses, owing to the relatively equivalent transcript accumulation in the SAM-proper and P0/P1domains. In support of this hypothesis, the SAM1.1 and SAM3.0 gene chips contain additional *ago1* co-orthologs that were not detected as differentially expressed in this analysis. It appears likely that as in *Arabidopsis*, the maize *ago1* gene family has expanded and paralogs became subfunctionalized to perform specialized tasks during leaf development and/or shoot meristem maintenance. Plant miRNAs are described that function in the ARGONAUTE1-directed regulation of leaf initiation and polarity, including miR166, and miR156 (reviewed in [73],[74]). Although these regulatory RNAs and/or their mRNA precursors are detected in both the SAM-proper and the initiating leaves of maize, mature microRNAs preferentially accumulate in the P0 and leaf primordia [75], which may be functionally correlated with the differential expression of one or more of the *ago1*-co-orthologs identified herein.

We speculate that the SAM-upregulated expression of a maize *ago4-like* ^35^ gene, predicted to function during regulation of siRNA-induced gene silencing and maintenance of DNA methylation [46],[47], may be elicited in response to the pronounced upregulation of retrotransposon transcription that is observed in the maize SAM [25]. Moreover, six genes predicted to function during DNA repair are upregulated in the SAM-proper versus just two in the P0/P1 (Figure 2), which may reflect selective pressures to maintain a mutation-free DNA template in the indeterminate, stem cell population of the meristem. Ultimately, the SAM is the source of all the somatic cells comprising the plant shoot, as well as the germinal cells within floral organs. While DNA repair is certainly occurring in the P0/P1, spontaneous mutations in the DNA of sterile, determinate leaf primordia may be subjected to weaker selective pressure as compared to the SAM.

Of the 66 protein fate genes upregulated in our microarray analyses, 42 were identified in the SAM-proper (Figure 2). Although ubiquitination and additional mechanisms of proteolysis are widespread throughout plant tissues, these data suggest the particular importance of these proteolytic pathways during SAM function. Previous studies in *Arabidopsis* and rice revealed that 26S proteaosome-dependent proteolysis is required for shoot meristem maintenance and identity [76]–[79]. Our data suggest that 26S proteaosome-dependent proteolysis is also important during the function of the maize SAM, and likewise implicates ubiquitin-related proteases, serine carboxy peptidases, OTU-like cysteine proteases, CLP proteases, aspartic proteases and various SUMO proteins during SAM function (Table S1).

Multiple categories of gene function are identified as upregulated during leaf initiation (Figure 2). Genes involved in cell wall biosynthesis and cell division/growth are logically co-regulated, and both gene categories are significantly upregulated in the P0/P1. Although it is true that cell division is absolutely required in the CZ of the SAM in order to replace cells lost during organogenesis and to maintain the meristematic stem cell population, live imaging in *Arabidopsis* has shown that mitotic activity in the PZ during leaf initiation is more expansive and proceeds at a faster rate than in the SAM proper [80]. Therefore, our array data are in agreement with both classical (reviewed in [81]) and recent descriptions of differential cell division rates within SAM functional zones. Although the maize *yabby-like* ^8–10^ genes upregulated in the P0/P1were placed in the separate GO category of transcription, the YABBYs are likewise presumed to function during expansive organ growth [17],[26],[82].

A maize homolog (*Zm-grf1* ^6^) of a family of transcription factors that regulate cell expansion in *Arabidopsis* leaves and cotyledons [30] was also identified in the P0/P1 dataset (Table 1). Bioinformatic analyses reveal that *grf1* homologs in *Arabidopsis* and rice have complementary target sites for miR396, a relatively rare small RNA that is either expressed at very low levels or in a limited number of cells/tissues [83]. *Zm-grf1* is expressed in the SAM periphery (Figure 3D) and leaf primordia and is downregulated in leaf-arrested SAMs (Figure 4), implicating a function very early in maize leaf development. *Zm-grf1* also harbors the conserved miR396 recognition motif, and thus represents an intriguing candidate gene for reverse genetic analyses of microRNA-regulated leaf development. The *grf* genes function redundantly in *Arabidopsis* [30] and at least eight *grf1*sequence paralogues are present in maize, suggesting that reverse genetic analyses utilizing RNAi approaches or miR396-resistant transgenes may be more informative than characterization of *Zm-grf1* knockout alleles.

Nearly three times as many gene elements involved in chromatin structure and remodeling were upregulated in the P0/P1 as in the SAM-proper (45 versus 16). These data may reflect the fundamental and widespread changes in chromatin that are predicted to accompany the switch from meristematic to leaf developmental programs. Alterations in chromatin structure are inherent when changing from the propagation of an extant developmental state (i.e. the SAM) to the installation of a new developmental program (i.e. leaf initiation), and may be further enhanced during the transition from an indeterminate to a determinate developmental field.

### Reverse Genetic Analyses Reveal Differences in the Evolution of the *D-cyclin* Gene Family in Maize and *Arabidopsis*

Previously uncharacterized in maize, the *asc1* gene was selected for reverse genetic analysis because our microarray and in situ hybridization analyses revealed significantly upregulated expression in leaf primordia (Table S1; Figure 3F). Moreover, the related genes *Zm-cycD4* and *Zm-cycD2* are also contained on the SAM 3.0 gene chip used in these assays, although neither gene was identified as differentially-expressed. This failure to detect redundant, differential expression of D4-CYCLIN paralogs in the maize SAM suggested that potential mutant phenotypes conferred by *asc1* mutations may not be masked by paralogous gene functions.

As shown in Figure 6, single mutations in *asc1* condition infertile plants with extreme reductions in leaf width and plant height. Interestingly, these mutant phenotypes are more widespread and severe than those observed in *Arabidopsis* triple mutant plants homoyzygous for mutations in each of three D3-cyclin paralogs [64], which are phylogenetically distinct from the CD4/CD2 cyclins (Figure 5C). In addition, whereas mutations in each of the paralogous *D4-cyclin Arabidopsis* genes condition mild reductions in hypocotyl stomatal number [65], solo asc1 mutants exhibit profound abnormalities in leaf stomatal patterning (Figure 6). These asc1 mutant phenotypes suggest that ASC1 is required for normal maize leaf development, and that subfunctionalization of *D-cyclin* gene function has proceeded quite differently within the maize and *Arabidopsis* lineages. These data further demonstrate that laser microdissection-microarray analysis is a tractable approach toward the identification of important gene functions within adjacent yet distinct microdomains during maize shoot development.

## Materials and Methods

### Plant Materials

Seedlings of the maize inbred B73 were raised in a growth chamber on a 15 hr light cycle. Samples were incubated at 25°C during the light cycle and 20°C during the dark cycle. Seedlings were harvested for dissection and fixation at 14 days after germination.

For use in shoot-apex culture, maize shoot apices were hand-dissected from 14-day-old seedlings to remove all except the four youngest leaf primordia as described [4]. Dissected apices were cultured on maize culture medium (MCM; described in [4]) containing 30 µmol N-1-naphthylphthalamic acid (NPA) dissolved in DMF, or in maize tissue culture media containing equal amounts of DMF but no NPA. Apices were incubated for 14 days on a 14 hour light cycle at 28°C (light period) or 24°C (dark period).

### Genetic Analyses

EST clone AW067338 was used to identify maize core gene AC196112.3_FG024 located at location 39,743–41,597 of contig 45 on chromosome 7 (Maize Genome Browser; http://www.maizesequence.org/index.html). As the second *D4 cyclin* gene characterized in maize, this locus was named *leaf cyclinD42* (*asc1*). For reverse genetic analyses of *asc1*, DNA samples were prepared from pooled F2 seedling progeny obtained via self-pollination of 3,456 F1 plants containing active *Mutator* (*Mu*) transposon systems and subjected to PCR-based screens using nested *asc1* gene-specific primers (*lcd1*-CTTGCATCCTCCACTTGAGC and *lcd2*-AGCAGCTGTGTCATCCAAGC) and a *Mu* specific primer (*MuTIR*-AGAGAAGCCAACGCCAWCGCCTCYATTTCGTC). To rule out false-positive results derived from multiple *Mu* insertions, control reactions were performed with the *Mu* primer only. PCR reactions with specific products only from the nested PCR amplifications were sequenced to verify the *Mutator* transposon insertion. Sibling seed from PCR-positive families were planted in a corn nursery in Aurora, NY, screened for developmental phenotypes and outcrossed for two generations to inbred B73. Interallelic crosses of plants heterozygous for independent *Mu*-insertion alleles of *asc1* failed to complement, indicating the mutant phenotype observed in F2 progeny of self-pollinated plants harboring *asc1-Mu* insertion alleles are due to mutations in *asc1*.

### Phylogenetic Analyses

Alignments were performed on protein sequences translated from ten *Arabidopsis* proteins AtCYCD1;1 (NM105689), AtCYCD2;1 (NM127815), AtCYCD3;1 (NM119579); AtCYCD3;2 (NM126126), AtCYCD3;3 (NM114867), AtCYCD4;1 (NM125940), AtCYCD4;2 (NM121082), AtCYCD5;1 (NM119926), AtCYCD6;1 (NM116565), AtCYCD7;1 (NM120289), a D1-CYCLIN from *Physcomitrella patens* (CAD32542), three CYCLINS from *Antirrhinum majus* including AmCYCD1 (AJ250396), AmCYCD3a (AJ250397) and AmCYCD3b (AJ250398), ASC1 and the maize CYCLINS ZmCYC2;1 (AF351189), ZmCYC4;1 (AF351191), ZmCYCD5;1 (AF351190) and ZmCYCD5;2 (AY954514). Sequences were aligned using CLUSTALX 2.0.4, and cladograms were generated with PAUP 4.0 using the Maximum Parsimony method and after treating gaps in the alignment as missing data. Equivalent cladograms were generated using the Neighbor Joining method and without removing gaps; bootstrapping values were calculated for 1,000 replicates.

### Histological Analyses

Maize seedlings harvested at 14 days after germination were fixed in FAA, paraffin-embedded, sectioned at 10 µm, and stained in either Toluidine Blue O or Safranin-Fast Green using Johanssen's method as described [84]. Immunohistochemical analyses of KNOX protein accumulation were performed as described [13]. Epidermal images were obtained using cyanoacrylate glue surface impressions as described [85]. All micrographs were imaged on a Zeiss Z1-Apotome microscope (Thornwood, NY).

### Laser Microdissection/RNA Extractions of SAM Domains

Seedling shoots were fixed by incubating in acetone, paraffin-embedded as described and sectioned at 10 µm as described [25],[26]. All laser-microdissections were performed using a P. A. L. M. Laser Microbeam (P.A.L.M. Microlaser Technologies, Bernried, Germany). SAM tissue domains were captured from 5–10 sections per sample, comprised of 0.3 mm^2^–2 mm^2^ of tissue. Six biological replicate samples were obtained. RNA was isolated from laser-microdissected tissue as described [25],[26]; RNA amplifications were performed using the RiboAmp™ HS kit (Arcturus, Mountainview, CA) according to the manufacturers protocol.

### Microarray Hybridizations and Analyses

The SAM cDNA-enriched SAM1.1 and SAM3 microarrays used in these experiments were as described [26]. The MIAME guidelines utilized, hybridization protocols, and array scanning procedure were as described [25],[26]. All microarray data are available at Gene Expression Omnibus (GEO; http://www.ncbi.nlm.nih.gov/geo). Six biological replicate array hybridizations were performed. One of the six SAM1.1 slides was excluded from analysis due to poor hybridization quality and areas of very high background. Data from the other 11 slides were normalized within slides using loess normalization and across slides within each platform using scale normalization [86]. The limmaGUI R package [87] was used to conduct a linear model analysis for each gene following described methods [88]. The method of Benjamini and Hochberg [89] was used to estimate the false discovery rate associated with the identified sets of differentially expressed genes. Annotations of the predicted GO functions for all differentially expressed genes were performed as described [27]; annotated data is presented at SAM-The Maize Shoot Apical Meristem Project (http://sam.truman.edu/geneva/geneva.cgi).

EST sequences of the 1,312 microarray elements that were found to be differentially-expressed in the P0/P1 and SAM datasets were sorted by BLAST homology analyses to the contigs sequenced maize genomic DNA (i.e. maize contigs) in order to convert array elements (ESTs) into maize gene contig (MGC) groups. For multiple ESTs that hit a single MGC, the EST list was collapsed under the single MGC identity, and mean and standard error of mean (sem) were calculated for *P*-values and fold change of those ESTs. Sample size (n) value for mean and sem calculations is represented by the number of ESTs for a single MGC group. Multiple ESTs linked to a single MGC group were searched in both P0/P1 and SAM datasets to detect potential dual hits; such MGC groups containing P0/P1- and SAM-specific ESTs were removed from the dataset. To this end, 10 MGC groups representing 20 P0/P1- and 9 SAM-specific ESTs were removed. In some cases, a MGC could not be assigned for an EST, as indicated by the identifier ‘contig:NONE’. In other cases, single or multiple ESTs hit multiple MGCs (Table S1).

A test based on the binomial distribution was used to identify functional categories for which the discrepancy between the number of genes upregulated in SAM and the number upregulated in P0/P1 was significant. Conditioning on n = total number of genes identified in a given category, the null hypothesis that the proportion of genes upregulated in SAM was equal to 1/2 was tested against the alternative that the proportion was not 1/2. A p-value was obtained by comparing the observed number of genes upregulated in SAM to a binomial distribution with n trials and success probability 1/2. For example, given that 34 genes predicted to function in RNA binding were identified as differentially expressed, the probability that only 9 of these 34 would be up in SAM relative to P0/P1 is approximately 0.0045 according to a binomial distribution with 34 trials and success probability 1/2. This yields a two-sided p-value of 2*0.0045 = 0.009 and suggests that discrepancy (9 up in SAM vs. 25 up in P0/P1) cannot easily be explained by a simple chance mechanism.

### Transcript Analyses

qRT-PCR analyses of NPA-treated and untreated shoot apices (described in Plant Materials, above) were performed on cDNA prepared from tissue-cultured/laser-microdissected SAMs as described [55]. Analyses utilized three technical replications performed on pooled cDNA prepared from ten microdissected SAMs. Gene-specific primer pairs used in these analyses were as follows: AI855049 (5′-CAGAATCATCACCTACACCT-3 and 5′-GAGTAGTAGAAGATTGCTGTGAG-3′); DN220821(5′-GCTAATGAGCATAGTATGCC-3′ and 5′-CTGCTCATTACCATGTCCTG-3′); CD527823 (5′-TCCGTCTTGTACATGTGAG-3′ and 5′-TCTCGACATTCTTAAGGAGC-3′); CD670256 (5′-GGTCTCTAAAGTCACTGAAACC-3′ and 5′-GAGCTGATCCCTTAGTTAAGTC-3′); BG840831 (5′-GATCAAATCATAGACCTAGAGTCC-3′ and 5′-ATTGGTGTAGTTTCCTAGCTG-3′); AY313902 (5′ CCTCAAGAAGACCTTCAAGAC-3 and 5′-TTATTAGAATGGAGTGATGCCC-3′); CB380920 (5′-TCACCGTCAGAATTTACGTC-3′ and 5′-GCATAAACAACCACTGAACC-3′); CA998660 (5′- TTGAACTCATCCGCTTTCTC-3′ and 5′-TTGACACATTCCGTCTACAG-3′); BM073971 (5′-CCTCAAGGCATTCAGATCTC-3′ and 5′-AGATGATGTCTTCCTGTCGT-3′).

Fourteen-day-after-germination maize B73 seedlings were processed for in situ hybridization as described [90] with modifications [91]. Gene-specific probes were synthesized from the cDNA clones DN229322, BM073398, CB381076. BG840831, AW067338. For each gene-specific probe analyzed, at least six replicate samples were hybridized.

For use in RNA gel-blot hybridizations, total RNA was extracted from 14-day seedlings using the Trizol lysis method and prepared for Northern transfer as described [92]. The gene-specific *asc1* probe was prepared from genomic DNA using the primer pair 5′-CGGTTTCCTGGAGTCTGAGG-3′ and 5′- CTTGCATCCTCCACTTGAGC-3′, which amplifies a 776 bp fragment spanning exons 1–4 (Figure 5A).

## Supporting Information

Figure S1 Transcript analyses of *asc1* mutant alleles.
(1.56 MB TIF)

Table S1 Genes differentially expressed in SAM functional domains.
(0.23 MB XLS)

Dataset S1 Accession numbers of maize genes cited as superscripted numerals in this manuscript.
(0.04 MB DOC)
