# Supplementary figures and images for: Microdissection of Shoot Meristem Functional Domains
Source: PLoS Genet. 2009 May 8;5(5):e1000476. doi: 10.1371/journal.pgen.1000476 (PMC2673047; doi:10.1371/journal.pgen.1000476)

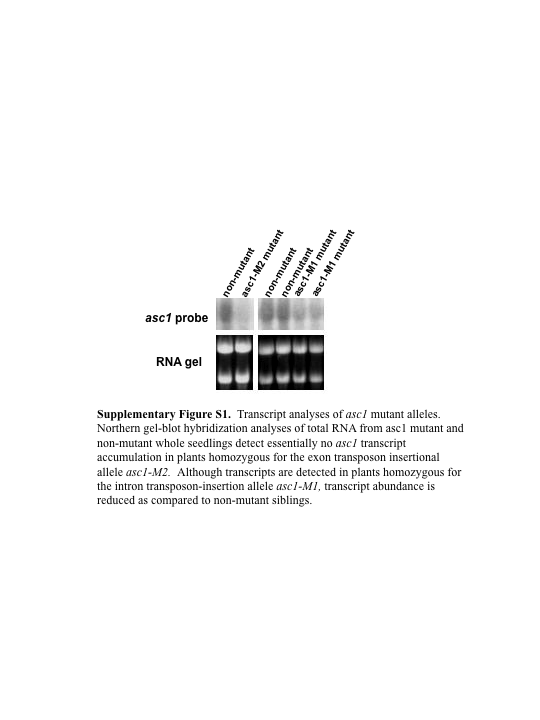

Supplement: Figure S1 — Transcript analyses of asc1 mutant alleles. (1.56 MB TIF) [file pgen.1000476.s001.tif]
