# Supplementary material for: Microdissection of Shoot Meristem Functional Domains
Source: PLoS Genet. 2009 May 8;5(5):e1000476. doi: 10.1371/journal.pgen.1000476 (PMC2673047; doi:10.1371/journal.pgen.1000476)
Supplement: Dataset S1 — Accession numbers of maize genes cited as superscripted numerals in this manuscript. (0.04 MB DOC) [file pgen.1000476.s003.doc]

**Supplementary File 1: Accession Numbers.**

Accession numbers of maize gene contigs (MGCs) corresponding to maize cDNA clones spotted on SAM1.1 and SAM3.0 microarrays and cited as superscripted numerals in this manuscript:

^1^AC191426.2-Contig19; ^2^AC214821.2-Contig11; ^3^AC200561.4-Contig49; ^4^AC183520.3-Contig25; ^5^AC185600.3-Contig17; ^6^AC190645.3-Contig32, AC207330.2-Contig32; ^7^AC204518.4-Contig32; ^8^AC195538.2-Contig93 ^9^AC202451.2-Contig53; ^10^AC194098.3-Contig35; ^11^AC210607.2-Contig20; ^12^AC177947.2-Contig69; ^13^AC204864.2-Contig55; ^14^AC190835.3-Contig124; ^15^ AC200180.3-Contig22; ^16^ AC197900.3-Contig51; ^17^ AC189039.3-Contig78; ^18^AC184701.4-Contig60; ^19^ AC194417.2-Contig101, ESTs DV549372 and CA829732 - no MGC identified; ^20^ AC202956.3-Contig71 and AC212441.2-Contig33; AC177945.3-Contig33; ^21^AC190571.2-Contig30; ^22^AC216353.1-Contig121; ^23^AC195895.2-Contig67; AC202564.2-Contig15; ^24^AC193985.3-Contig30; AC198380.3-Contig32; ^25^AC196112.3-Contig45; ^26^AC204359.2-Contig106; AC211925.2-Contig34; ^27^AC204879.2-Contig54; ^28^AC200258.3-Contig13; AC210021.3-Contig21; ^29^AC191627.3-Contig74; ^30^AC201833.4-Contig185; AC203943.2-Contig12; ^31^AC210021.3-Contig21; ^32^AC214136.2-Contig58; ^33^AC209206.2-Contig44; ^34^ AC210271.2-Contig15, contig:AC214136.2-Contig58; ^35^AC186578.3-Contig14; ^36^AC190972.4-Contig32; ^37^AC206901.3-Contig30; ^38^AC211363.2-Contig77; AC191640.3-Contig20; ^39^AC191640.3-Contig20; ^40^AC206827.2-Contig149; AC208082.2-Contig22; ^41^AC196679.4-Contig92; ^42^AC215810.2-Contig34; ^43^AC190694.3-Contig78; AC196125.3-Contig78; AC203914.2-Contig125; ^44^ EST DN214604, no MGC identified; ^45^AC177913.4-Contig119; ^46^AC204716.2-Contig21; ^47^AC211276.2-Contig22; ^48^AC191429.3-Contig28; ^49^EST DN221539 - no MGC; ^50^AC194132.3-Contig33; ^51^AC214507.2-Contig127; ^52^AC183950.1-Contig101; ^53^AC196297.3-Contig89; AC211194.2-Contig40; ^54^AC199481.3-Contig39; AC214830.2-Contig140; ^55^AC213879.2-Contig23; ^56^ontig:AC200198.3-Contig63; AC209370.2-Contig20; AC216888.1-Contig18; ^57^AC216888.1-Contig18; ^58^AC190986.2-Contig17; AC205697.2-Contig18; ^59^EST DY402450 - no MGC; ^60^ AC194323.2-Contig18; AC204211.2-Contig32; ^61^AC191049.3-Contig65; ^62^AC210422.2-Contig52; ^63^AC195324.2-Contig84; ^64^AC203318.2-Contig64; AC216879.1-Contig36; ^65^AC190927.4-Contig29; ^66^AC190503.1-Contig193; ^67^P0/P1 paralogs: AC206938.2-Contig21; AC215177.2-Contig12; EST BM072973, no MGC identified; ^68^SAM paralogs: AC190568.3-Contig17; AC210022.2-Contig39; AC190522.1-Contig103; ^69^P0/P1 paralogs: AC186428.3-Contig33; AC194605.2-Contig46; AC177886.2-Contig446; AC201914.3-Contig108; AC216881.1-Contig38; AC190762.3-Contig126; AC196961.2-Contig29; AC203785.3-Contig30; AC209366.2-Contig130; AC177886.2-Contig430; AC200255.2-Contig10; ^70^SAM paralogs: AC193447.4-Contig21,AC199873.4-Contig29; ^71^P0/P1 paralogs: AC209454.2-Contig77; AC193789.2-Contig44; AC210293.2-Contig17; AC215229.2-Contig159; AC195359.3-Contig32; ^72^SAM paralogs: AC190835.3-Contig124; AC204645.2-Contig101; AC195860.2-Contig17; AC206279.3-Contig30; AC190842.3-Contig52, AC203950.2-Contig12; AC214115.2-Contig64; AC210831.2-Contig77, AC215991.2-Contig75; AC198688.2-Contig46; ESTs DN228146 and BM338990, no MGC identified; ^73^P0/P1 paralog: AC206992.2-Contig20; ^74^SAM paralog: AC182605.4-Contig32; AC191293.3-Contig60; ^75^P0/P1 paralog: AC204765.2-Contig46; ^76^SAM paralog: AC185641.3-Contig41; ^77^AC205557.2-Contig43; ^78^AC198290.2-Contig37; ^79^AC194970.3-Contig28; ^80^AC204086.2-Contig34, AC206833.2-Contig38; ^81^AC191550.2-Contig31.
